# Prehospital Videolaryngoscope Availability and Use in Belgium: A Nationwide Cross-Sectional Survey

**DOI:** 10.7759/cureus.117841

**Published:** 2026-10-13

**Authors:** Georgios Wallden, Stefano Malinverni

**Affiliations:** 1 Emergency Department, CHU Saint-Pierre, Brussels, BEL

**Keywords:** airway management, emergency medical services (ems), intratracheal intubation, prehospital intubation, video laryngoscopy (vl)

## Abstract

Background

Emergency endotracheal intubation (ETI) outside the operating room is challenging because of patient-, operator-, situational-, and logistical factors. Videolaryngoscopy is an alternative to direct laryngoscopy and can reduce ETI failure and complications. In Belgium, no published data are available regarding the availability of videolaryngoscopes (VL) for prehospital ETI by mobile intensive care unit (MICU) crews. The main objective of this survey was to estimate VL availability among responding Belgian MICUs. The secondary objective was to identify physicians' prehospital VL use patterns as well as to explore factors potentially associated with VL use.

Methods

This cross-sectional survey used two online questionnaires distributed to all Belgian MICUs. Questionnaire A was intended for the heads of department and collected information about VL availability and local protocols. Questionnaire B was intended for all MICU physicians and assessed their frequency of VL use and their reasons for using it or not using it.

Results

Of the 96 eligible Belgian MICUs, 35 heads of department responded to Questionnaire A (36.5%) and 32/35 (91.4%; 95% confidence interval, CI, 77.6-97.0%) reported VL availability. Out of the 115 MICU physicians who responded to Questionnaire B, 81 reported using a VL (70.4%; 95% CI, 61.5-78.0%). Among respondents with access to a VL, reported use differed by region of practice (p = 0.012); odds of VL use were lower in the Brussels-Capital Region (odds ratio 0.123; 95% CI, 0.028-0.546). More than half of the VL users reported using it for less than 10% of their ETIs, and only 6.2% used it as a first-choice device. The most frequently reported indication for VL use was failed ETI with a direct laryngoscope (81.5%).

Conclusion

VL availability was high among responding Belgian MICUs. However, the reported prehospital VL use was infrequent.

## Introduction

Endotracheal intubation (ETI) is an advanced airway management procedure aimed at securing the airway and establishing an interface for invasive mechanical ventilation. It is routinely performed in the operating room (OR) in patients undergoing general anesthesia, but it also has numerous indications in the emergency department (ED), including acute respiratory failure, inability to protect the airway, severe traumatic brain injury, and following cardiac arrest [1,2]. ETI performed outside the OR is recognized as challenging because of patient-related, situational, logistical, and operator-related factors. Outside the OR, emergency ETI is considered difficult or requires multiple attempts in up to 13.2% of cases and may result in complications, the most common of which are hypoxia, arterial hypotension, esophageal intubation, and aspiration [3]. In the prehospital setting, environmental factors may further complicate ETI attempts. These include patient positioning (lying on the ground or trapped inside a vehicle), lighting conditions, and weather conditions [4].

An alternative to conventional direct laryngoscopy is videolaryngoscopy. A Cochrane meta-analysis compared ETI using direct laryngoscopes (DL) with three types of videolaryngoscopes (VL) (Macintosh-style, hyperangulated, and channeled), regardless of the clinical setting (elective or emergency, in-hospital or prehospital), and demonstrated a reduction in intubation failure rates with VLs. The relative risks of ETI failure were 0.41 (95% confidence interval, CI, 0.26-0.65) with Macintosh-style VLs, 0.51 (95% CI, 0.34-0.76) with hyperangulated VLs, and 0.43 (95% CI, 0.30-0.61) with channeled VLs [5]. According to the Difficult Airway Society (DAS), VL “should be considered as an option for any intubation of a critically ill patient” [6]. More recently, the Spanish Societies of Anesthesiology, Resuscitation and Emergency Medicine, as well as the Latin American Federation of Emergency Medicine, have recommended VL as the universal first-line tool for ETI, ahead of DL [7].

Specifically in the prehospital setting, the German Guideline for Prehospital Airway Management defines the use of a Macintosh-style VL as the new standard of care [8]. Despite these recommendations, 55% of physicians in Germany still use a DL as their first-choice technique for prehospital ETI (PHETI) [9]. In Belgium, in the absence of similar national recommendations, no published data are available regarding the actual availability or use of VLs for PHETI by physicians working in mobile intensive care units (MICU).

The primary objective of this survey was to estimate VL availability among responding Belgian MICUs. The secondary objective was to assess physicians’ use of VLs for emergency adult intubation in the prehospital setting and to explore factors potentially associated with their use or non-use.

## Materials and methods

Study design

This nationwide cross-sectional survey used two electronic questionnaires distributed to healthcare professionals working in Belgian EDs. The study was approved by the Institutional Ethics Committee of the CHU Saint-Pierre, Brussels, Belgium (Approval No. B0762026260105, dated January 14, 2026). As the questionnaire responses were anonymous and did not contain any personal data, they did not fall within the scope of the European General Data Protection Regulation (GDPR). Nevertheless, explicit consent was obtained from all respondents for their responses to be used for the purposes of this study. This study adhered to the Strengthening the Reporting of Observational Studies in Epidemiology (STROBE) recommendations for observational studies and to the Consensus-Based Checklist for Reporting of Survey Studies [10,11].

Population and questionnaires

Belgian MICU teams consist of a physician and a nurse and are affiliated with an accredited hospital ED [12]. The list of hospitals with a MICU service is publicly available from the Belgian Federal Ministry for Public Health, Food Chain Safety and Environment [13].

Questionnaire A was intended for all heads of EDs with a MICU in Belgium (Table 1). Questionnaire B was intended for all physicians working in a MICU in Belgium (Table 2). These two questionnaires were newly developed by the authors. Both questionnaires were created using Google Forms (Google LLC, Mountain View, California, United States), and their access links were sent by email to the relevant EDs, either to the head of department or to the department’s administrative office, depending on the publicly available contact information. The email requested that Questionnaire A be completed by the head of department and that Questionnaire B be forwarded to all physicians working in the MICU. The questionnaires were available online from January 18, 2026 to February 28, 2026, in three languages (French, Dutch, and English).

**Table 1:** Questionnaire A (for heads of department) Responses to all questions were mandatory. ED: emergency department; MICU: mobile intensive care unit; N/A: not applicable; PHETI: prehospital endotracheal intubation; VL: videolaryngoscope.

Section	Question	Type of response	Response options
Introduction	Language of the form	Select one	English
			Dutch
			French
	I agree that my responses may be used for this study	Select one	Yes
Your center	Region	Select one	Brussels-Capital
			Flanders
			Wallonia
	Type of hospital	Select one	Academic
			University
			General public
			General private
			Other
	MICU at your hospital	Select one	Permanently
			Shared with other hospitals
	Number of MICU missions per year	Select one	< 1500
			1500-3000
			> 3000
	Main physician on board the MICU	Select one	Always a specialist
			Always a resident
			Specialist or resident
			I don't know
	Use of VL in the ED	Select one	Always
			Often
			Sometimes
			Rarely
			Not available
Pre-hospital use of VL	VL available on board the MICU	Select one	Yes
			No
	Type of VL	Free-text field	N/A
Department protocol	Recommendation to use VL as first-line in the pre-hospital setting	Multiple responses allowed	No department protocol
			Yes, systematic first-line use of VL
			Yes, use of VL at the operator's discretion
			Yes, first-line use of VL for severely injured patients
			Yes, first-line use of VL for patients in cardiac arrest
			Yes, use of VL in case of failed intubation with a direct laryngoscope
			I don't know
Training	Formal training in the use of VL organized by the department for MICU physicians	Select one	No
			Yes, supervised by ED physicians
			Yes, supervised by physicians from other departments
			I don't know
			Other
Data collection	Organized data collection on PHETI to assess the first-attempt intubation success rate and complications	Select one	Yes
			No
			I don’t know
Other	Would you like to provide us with any additional information or comments?	Free-text field	N/A

**Table 2:** Questionnaire B (for MICU physicians) Responses to all questions were mandatory. DL: direct laryngoscope; MICU: mobile intensive care unit; N/A: not applicable; PHETI: prehospital endotracheal intubation; VL: videolaryngoscope.

Section	Question	Type of response	Response options
Introduction	Language of the form	Select one	English
			Dutch
			French
	I agree that my responses may be used for this study	Select one	Yes
Demographics	Age	Select one	< 31
			31-40
			41-50
			51-60
			> 30
	Gender	Select one	Female
			Male
			Prefer not to answer
			Other
	Initial specialty training	Select one	Ongoing
			Completed
			None
	Initial specialty	Free-text field	N/A
	Do you hold the Certificate in Acute Medicine?	Select one	Yes
			No
	Additional specialty training	Select one	Ongoing
			Completed
			None
	Additional specialty	Free-text field	N/A
	Place of specialty training	Select one	Belgium (French-speaking Community)
			Belgium (Dutch-speaking Community)
			Outside Belgium
Your center	Region	Select one	Brussels-Capital
			Wallonia
			Flanders
	Type of hospital	Select one	Academic
			University
			General public
			General private
			Other
MICU shifts	Type of shifts	Select one	Daytime only
			On-call shifts only
			Daytime and on-call shifts
	Number of years of MICU experience	Free-text field	N/A
	Use of VL for PHETI	Select one	Yes
			Never
VL for PHETI: Never	Reasons	Multiple responses allowed	No VL available on board my MICU
			I have never needed an alternative to DL
			I do not feel comfortable using a VL
			I have never been trained in the use of a VL
			Other
VL for PHETI: Yes	Percentage of PHETIs performed with a VL	Select one	< 10%
			10-25%
			25-50%
			50-75%
			75-90%
			> 90%
			I don’t know
	Reasons	Multiple responses allowed	Always as first-line
			Depending on patient-related risk factors for difficult intubation
			In case of failed intubation with DL
			Always as first-line for severely injured patients
			Always as first-line for patients in cardiac arrest
			Always as first-line for severely burned patients
			Other
Training	Have you received training in the use of a VL?	Multiple responses allowed	Never
			Yes, organized by my department
			Yes, organized by external organizations
			Yes, during my specialty training
			Other
Other	Would you like to provide us with any additional information or comments?	Free-text field	N/A

Collected data

Data collected through Questionnaire A (heads of department) included geographic and operational information about the MICU, the availability of a VL onboard (defined as a yes/no response indicating whether a VL was available onboard), and the presence of departmental protocols regarding specifying clinical situations in which VL was to be used. Questionnaire B (MICU physicians) collected demographic data, information on the frequency of prehospital VL use (defined as the percentage of PHETIs performed using a VL, categorized as never, <10%, 10-25%, 25-50%, 50-75%, 75-90%, or >90%), motivations for and barriers to its use, and training in VL use (whether physicians had received formal training and, if so, in what setting). As responses to all questions on the forms were mandatory, all questionnaires were complete, and there were no missing data. Because responses to both questionnaires were anonymous, duplicate responses could not be ruled out. Anonymity also precluded the collection of information on nonresponding centers and physicians and prevented reminders from being sent.

Outcomes

The primary outcome was the proportion of responding MICUs reporting a VL available onboard. Secondary outcomes were the proportion of responding MICU physicians who reported using a VL and the exploration of demographic, institutional, and educational factors potentially associated with VL use.

Statistical analysis

Continuous data were expressed as median (interquartile range), whereas categorical data were expressed as number (percentage), with 95% CI for proportions calculated using the Wilson method. Continuous variables were compared using the Wilcoxon-Mann-Whitney test, as the assumption of normality was not met based on the Shapiro-Wilk test. Categorical variables were compared using Fisher’s exact test for 2 x 2 contingency tables and the exact Freeman-Halton extension for larger contingency tables. Analyses of factors potentially associated with VL use were restricted to respondents who reported access to a VL.

Given the significant difference in physicians’ region of practice between VL users and non-users, a post-hoc analysis was performed comparing the Brussels-Capital Region with the Flemish and Walloon Regions combined, using Fisher’s exact test.

Because responses to Questionnaire B were anonymous, the respondents’ centers could not be identified. Therefore, within-center clustering could not be accounted for in the analysis.

A p-value < 0.05 was considered the threshold for statistical significance. All statistical analyses were performed using R software (version 4.3.3; http://cran.r-project.org; R Foundation for Statistical Computing, Vienna, Austria).

## Results

Baseline characteristics

Among the 118 specialized EDs in Belgium, 96 have a MICU. The heads of 35 of these departments responded to Questionnaire A (36.5%). The response rate was six out of nine in the Brussels-Capital Region (66.7%), 13/37 in the Walloon Region (35.1%), and 16/50 in the Flemish Region (32.0%) (Figure 1). 

**Figure 1:**
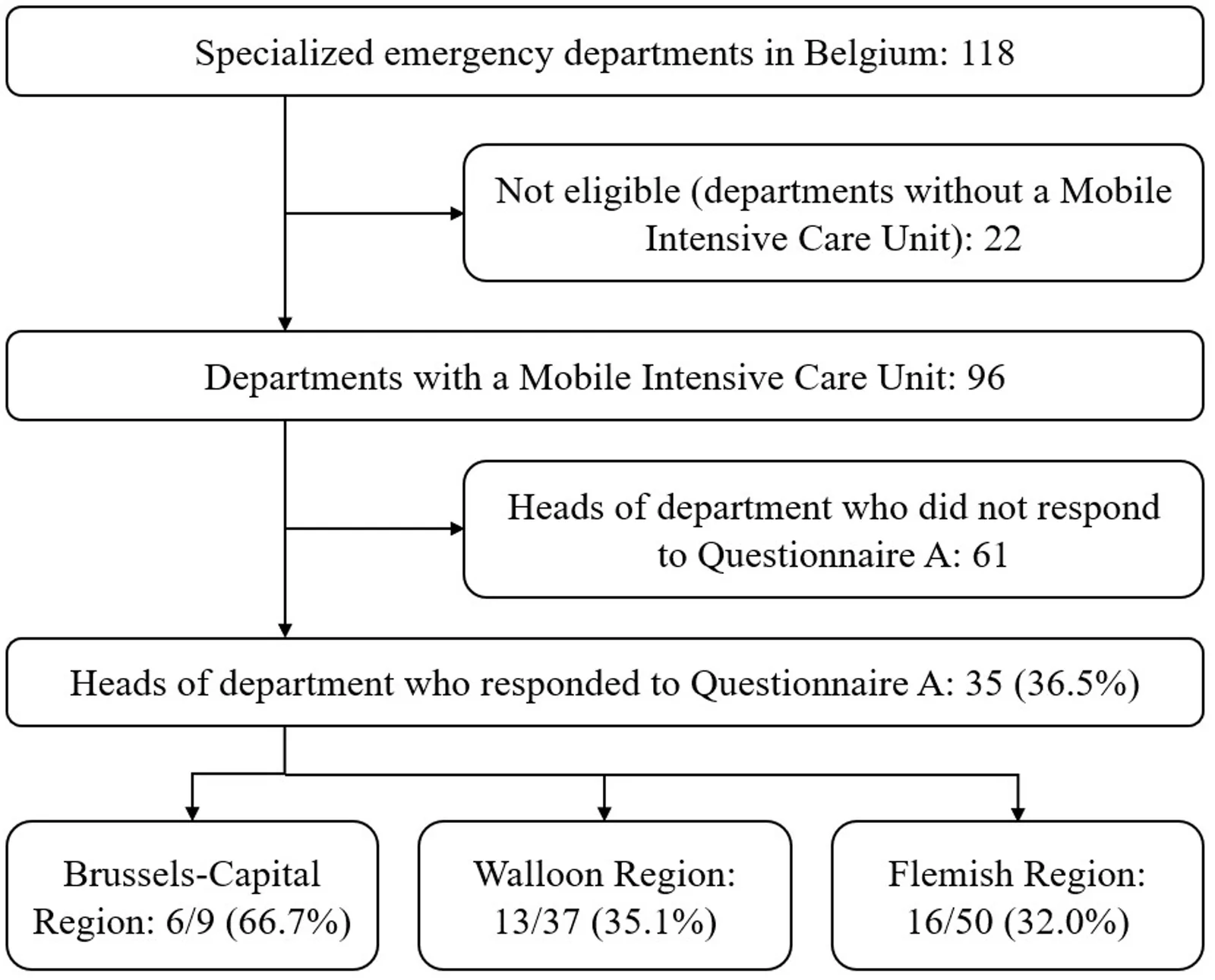
Inclusion flowchart for questionnaire A

Responses were obtained from public general hospitals (12/35, 34.3%), private general hospitals (9/35, 25.7%), and academic or university hospitals (14/35, 40.0%) (Table 3).

**Table 3:** Prehospital videolaryngoscope availability (questionnaire A) Categorical variables are presented as number (percentage). ED: emergency department; MICU: mobile intensive care unit; VL: videolaryngoscope.

Characteristic	All answers	Available VL
n	35 (100.0)	32/35 (91.4)
Region		
Brussels-Capital	6 (17.1)	5/6 (83.3)
Flanders	16 (45.7)	14/16 (87.5)
Wallonia	13 (37.1)	13/13 (100.0)
Hospital type		
Academic or University	14 (40.0)	12/14 (85.7)
General public	12 (34.3)	12/12 (100.0)
General private	9 (25.7)	8/9 (88.9)
MICU missions		
< 1,500 per year	11 (31.4)	11/11 (100.0)
1,500-3,000 per year	17 (48.6)	16/17 (94.1)
> 3,000 per year	7 (20.0)	5/7 (71.4)
VL available inside the ED		
Unavailable	1 (2.9)	1/1 (100.0)
Rarely/sometimes used	21 (60.0)	19/21 (90.5)
Often/always used	13 (37.1)	12/13 (92.3)

Questionnaire B was completed by 115 MICU physicians in Belgium. Because the total number of eligible physicians is unknown, the response rate could not be determined. Most of the respondents were specialist physicians (88/115, 76.5%), and most were under 41 years of age (80/115, 69.6%). Most physicians were specialized in acute or emergency medicine (99/115, 86.1%), and the majority had received their training in the French-speaking Community of Belgium (76/115, 66.1%). Their median MICU experience was 6 (3-12) years (Table 4).

**Table 4:** Characteristics of questionnaire B respondents Categorical variables are presented as number (percentage), and the continuous variable as median (interquartile range). MICU: mobile intensive care unit; VL: videolaryngoscope.

Characteristic	All respondents	VL available in their MICU
n	115 (100.0)	90/115 (78.3)
Age		
< 41	80 (69.6)	66/80 (82.5)
41-50	16 (13.9)	11/16 (68.8)
> 50	19 (16.5)	13/19 (68.4)
Gender: female	57 (49.6)	43/57 (75.4)
MICU experience (years)	6 (3-12)	6 (3-12)
Professional status		
Resident	27 (23.5)	22/27 (81.5)
Consultant/specialist	88 (76.5)	68/88 (77.3)
Basic specialty		
Emergency or acute medicine	99 (86.1)	76/99 (76.8)
Anesthesia	8 (7.0)	6/8 (75.0)
Other	8 (7.0)	8/8 (100.0)
Specialty training location		
Belgium (Dutch-speaking)	39 (33.9)	29/39 (74.4)
Belgium (French-speaking)	76 (66.1)	61/76 (80.3)
Training in VL use	72 (62.6)	53/72 (73.6)
Hospital type		
Academic or University	70 (60.9)	48/70 (68.6)
General public	21 (18.3)	20/21 (95.2)
General private	24 (20.9)	22/24 (91.7)
Region of practice		
Brussels-Capital	35 (30.4)	22/35 (62.9)
Flanders	38 (33.0)	28/38 (73.7)
Wallonia	42 (36.5)	40/42 (95.2)

Videolaryngoscope availability

Among the 35 responding centers, 32 (91.4%; 95% CI, 77.6-97.0%) had a VL available onboard their MICU. Of these, 23/32 (71.9%; 95% CI, 54.6-84.4%) were hyperangulated VLs, six out of 32 (18.8%; 95% CI, 8.9-35.3%) were Macintosh-style VLs, and one out of 32 (3.1%; 95% CI, 0.6-15.7%) was a channeled VL. Among the 32 centers with a VL, 11 (34.4%; 95% CI, 20.4-51.7%) did not have a departmental protocol regarding its use. VL use at the operator’s discretion was recommended in 15 centers (46.9%; 95% CI, 30.9-63.6%), in cases of failed ETI with a DL in five (15.6%; 95% CI, 6.9-31.8%), and for patients with severe trauma in two (6.2%; 95% CI, 1.7-20.1%). No protocol recommended VL use in patients experiencing cardiac arrest. No VL training was provided in 18 of these centers (56.3%; 95% CI, 39.3-71.8%). Training was organized by the ED in 10 centers (31.3%; 95% CI, 18.0-48.6%) and by other departments in three (9.4%; 95% CI, 3.2-24.2%). Only one center (2.9%; 95% CI, 0.5-15.5%) systematically collected data on PHETI and associated complications.

Videolaryngoscope use

Among the 115 responding physicians, 81 (70.4%; 95% CI, 61.5-78.0%) reported using a VL in the prehospital setting. Among the 90 respondents (78.3%; 95% CI, 69.9-84.8%) who reported having a VL available in their MICU, the rate of use was 90.0% (95% CI, 82.1-94.6%). Of the 115 respondents, 72 (62.6%; 95% CI, 53.5-70.9%) reported having received training in VL use. Among physicians with access to a VL, the region of practice was the only demographic or institutional factor significantly associated with VL use (Brussels-Capital 72.7%, Flanders 96.4%, Wallonia 95.0%, p = 0.012). The post-hoc regional difference analysis showed a lower VL use in the Brussels-Capital Region compared with the Flemish and Walloon Regions combined (p = 0.006; odds ratio, OR, 0.123; 95% CI, 0.028-0.546). Neither age, sex, specialty, nor specific training in VL use showed a statistically significant difference (Table 5).

**Table 5:** Comparison of VL users and non-users among respondents with access to a VL Categorical variables are presented as number (percentage), and the continuous variable as median (interquartile range). † Fisher’s exact test (for categorical variables with 2 x 2 contingency tables); ‡ Fisher’s exact test with the Freeman-Halton extension (for categorical variables with 3 x 2 contingency tables); § Wilcoxon-Mann-Whitney test; — not applicable (Fisher’s exact test does not yield a test statistic); * p < 0.05. MICU: mobile intensive care unit; N/A: not applicable; VL: videolaryngoscope; W: Wilcoxon-Mann-Whitney test statistic.

Characteristic	Respondents with an available VL	Users	Non-users	Test statistic	p-value
n	90	81 (90.0)	9 (10.0)	N/A	N/A
Age				—	0.732 ‡
< 41	66	58 (87.9)	8 (12.1)		
41-50	11	11 (100.0)	0 (0.0)		
> 50	13	12 (92.3)	1 (7.3)		
Gender: female	43	39 (90.7)	4 (9.3)	—	1.000 †
MICU experience (years)	6 (3-12)	5 (3-12)	6 (4-7)	W = 355	0.903 §
Professional status				—	1.000 †
Resident	22	20 (90.9)	2 (9.1)		
Consultant/specialist	68	61 (89.7)	7 (10.3)		
Basic specialty				—	0.786 ‡
Emergency or acute medicine	76	67 (88.2)	9 (11.8)		
Anesthesia	6	6 (100.0)	0 (0.0)		
Other	8	8 (100.0)	0 (0.0)		
Specialty training location				—	0.262 †
Belgium (Dutch-speaking)	29	28 (96.6)	1 (3.4)		
Belgium (French-Speaking)	61	53 (86.9)	8 (13.1)		
Training in VL use	53	48 (90.6)	5 (9.4)	—	1.000 †
Hospital type				—	0.142 ‡
Academic or University	48	43 (89.6)	5 (10.4)		
General public	20	20 (100.0)	0 (0.0)		
General private	22	18 (81.8)	4 (18.2)		
Region of practice				—	0.012 ‡ *
Brussels-Capital	22	16 (72.7)	6 (27.3)		
Flanders	28	27 (96.4)	1 (3.6)		
Wallonia	40	38 (95.0)	2 (5.0)		

Among VL users, 44/81 (54.3%; 95% CI, 43.5-64.7%) reported using a VL in less than 10% of cases, whereas only four out of 81 (4.9%; 95% CI, 1.9-12.0%) used it in more than 90% of cases (Figure 2). The reported indications for VL use were as follows, with multiple answers allowed: first-line use in all cases for five out of 81 (6.2%; 95% CI, 2.7-13.6%), failed ETI with DL for 66/81 (81.5%; 95% CI, 71.7-88.4%), patient-related risk factors for difficult ETI for 48/81 (59.3%; 95% CI, 48.4-69.3%), severe trauma for 17/81 (21.0%; 95% CI, 13.5-31.1%), severe burns for 14/81 (17.3%; 95% CI, 10.6-26.9%), and cardiac arrest for four out of 81 (4.9%; 95% CI, 1.9-12.0%).

**Figure 2:**
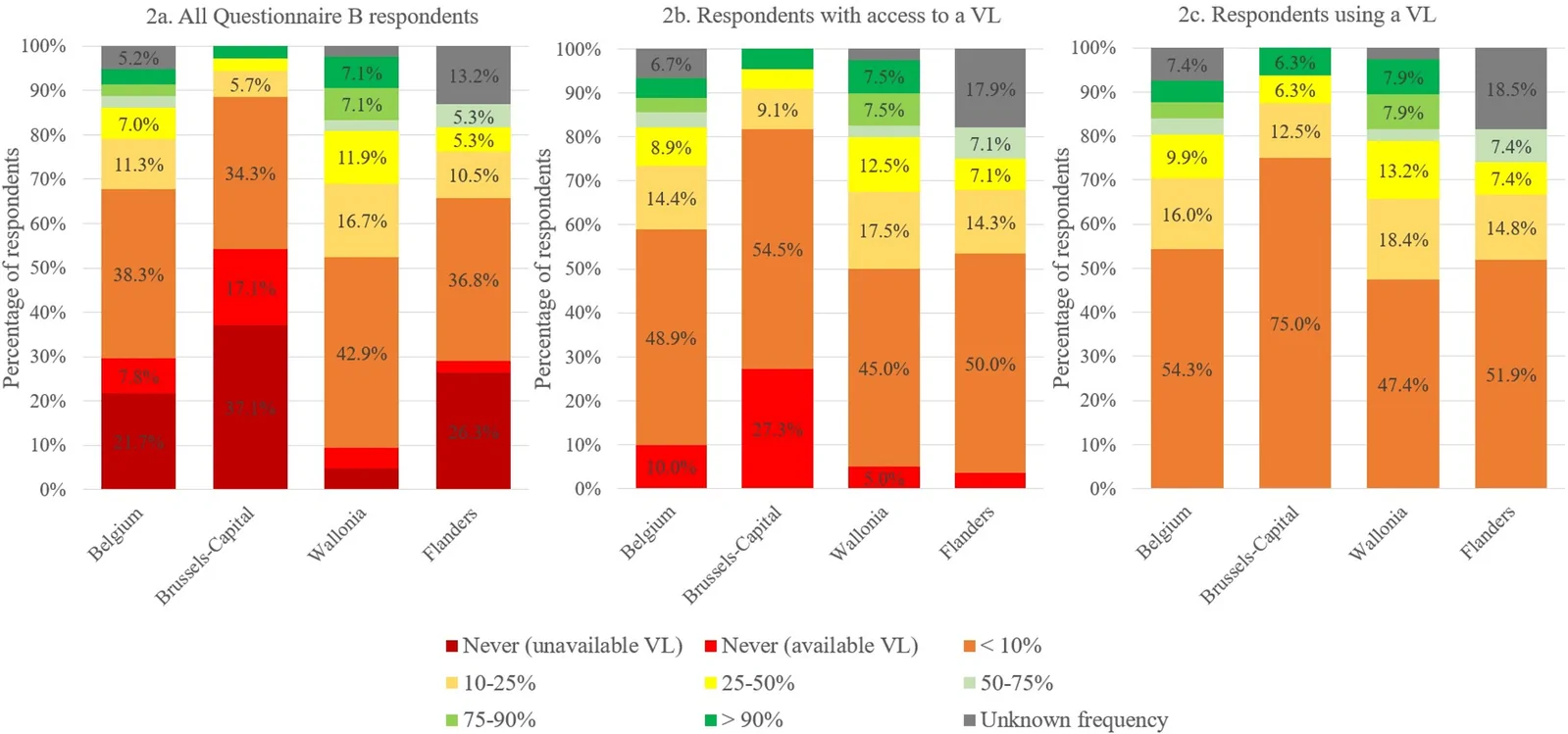
Frequency of prehospital videolaryngoscope use 2a. All Questionnaire B respondents. n = 115 (Brussels-Capital: 35; Wallonia: 42; Flanders: 38). 2b. Respondents with access to a VL. n = 90 (Brussels-Capital: 22; Wallonia: 40; Flanders: 28). 2c. Respondents using a VL. n = 81 (Brussels-Capital: 16; Wallonia: 38; Flanders: 27). VL: videolaryngoscope.

Among VL non-users, the main reason for non-use was the unavailability of a VL (25/34, 73.5%; 95% CI, 56.9-85.4%). Among those with access to a VL, the reported reasons for non-use (with multiple answers allowed) were the perception that an alternative to DL was unnecessary (eight out of nine, 88.9%; 95% CI, 56.5-98.0%), feeling uncomfortable using a VL (three out of nine, 33.3%; 95% CI, 12.1-64.6%), and lack of training in VL use (two out of nine, 22.2%; 95% CI, 6.3-54.7%).

## Discussion

The availability rate of VLs among the MICUs of the responding centers was 91.4%. Among the responding MICU physicians, 78.3% reported having access to a VL in the prehospital setting, and 90.0% of these physicians reported using it. VL was used as a first-line device by 6.2%, whereas the majority (54.3%) used it in fewer than 10% of their PHETIs. When comparing users and non-users, only the region of practice was significantly different (p = 0.012). The post-hoc analysis showed lower odds of VL use in the Brussels-Capital Region (OR 0.123).

Compared with neighboring countries, the availability of VLs in the prehospital setting in our study (91.4%) seemed to be higher than that reported in France and Germany but lower than that reported in Luxembourg. It should be noted, however, that the low response rate in our study limits the generalizability of this finding to Belgium as a whole. In France, a study comparable to the present one reported VL availability in 76.1% of MICUs, although it did not include firefighter medical response teams, which also constitute part of the prehospital emergency medical system [14,15]. In Germany, 45.2% of emergency response areas had equipped their MICUs with VLs in 2020, while acquisition was planned in an additional 41.1% of emergency response areas [16]. In Luxembourg, all MICUs are equipped with a VL [17].

In the absence of Belgian national recommendations specific to prehospital laryngoscope choice, 70.4% of responding MICU physicians reported using a VL, but the frequency of use was very low: more than half used it in fewer than one in 10 cases, and only 8.6% used it in more than half of cases. However, because the response rate is unknown, the representativeness of these findings at the national level cannot be assessed. These figures are comparable to those reported in France, where “66% of physicians have used a VL at least once” in the prehospital setting [14]. In Germany, where the latest guidelines consider VL the standard for PHETI, 44% of physicians use VL as their first-line technique, compared with 6.3% in our study [8,9].

The marked regional differences in prehospital VL use should be interpreted with caution given the exploratory nature of this post hoc analysis. However, these differences may also have been influenced by an overrepresentation of respondents from the Brussels-Capital Region. Indeed, 30.4% of respondents practiced in Brussels, whereas only nine out of 96 (9.4%) of MICUs are located there. This imbalance in the respondent sample may limit the representativeness of the overall estimate of VL use.

The reasons for VL use were heterogeneous but appeared to be more closely related to the technical difficulty of ETI (failed ETI with a DL or risk factors for difficult ETI) than to the patients’ clinical circumstances (trauma, burns, or cardiac arrest). Severe trauma was considered an indication for VL use by 21.0% of users, whereas, according to DAS recommendations, VL should systematically be used in cases of suspected cervical spine injury [18]. Furthermore, only 4.9% of users considered cardiac arrest an indication for VL use, although the 2025 European Resuscitation Council (ERC) guidelines state that VL use is “preferred” when it is immediately available [19]. This finding is consistent with the fact that none of the local protocols reported by participating centers mentioned cardiac arrest as an indication for VL use. However, neither the DAS nor the ERC guidelines distinguish between in-hospital and prehospital ETI.

Although the current literature supports the superiority of VL over DL in terms of first-attempt ETI success and reduction in complications, this has not yet been established in the specific context of PHETI. The few studies comparing VL and DL use for PHETI are characterized by substantial heterogeneity [2,7]. As this survey did not include any patient-centered outcomes, its findings cannot support recommendations regarding PHETI. Therefore, future research should focus on robust prospective studies to support the development of strong recommendations regarding the type of laryngoscope to use as a first-line device for PHETI.

Furthermore, should recommendations supporting VL use for PHETI be issued in Belgium, implementation would be logistically straightforward given the already high availability of VLs, but would require greater adaptation by departments and operators. Based on the reported reasons for non-use, more systematic training of practitioners should be considered, as only 40.6% of centers provide such training and only 62.6% of physicians report having received VL training. Another potential barrier to the widespread adoption of VL for PHETI may be financial, as the nomenclature of the Belgian National Institute for Health and Disability Insurance, which oversees national health insurance and reimbursement, does not distinguish between types of laryngoscopes. Hospital reimbursement is therefore provided as a fixed fee for the initiation of invasive ventilation by ETI [20]. The use of an electronic device such as a VL, which is more expensive than DL, may therefore represent an additional financial burden, potentially affecting hospital budgets. Although only a preliminary assessment of the barriers to current VL use is presented here, this issue could also be investigated in greater depth.

Finally, as part of a broader quality-of-care approach, the development of local protocols, which are still absent in more than one-third of centers, and the systematic evaluation of clinical practice, given that only one center reported collecting PHETI data, should be encouraged [21].

Study limitations

The main limitation of this study is the substantial risk of selection bias inherent to its design, which relied on voluntary responses. The high reported rate of VL availability may therefore be overestimated. The lower proportion of responding physicians who reported access to a VL is consistent with this possibility but does not establish it. Nevertheless, although these findings should be interpreted with caution, this survey provides valuable information, as it is the first study to explore VL availability in Belgium. The diversity of responding centers across all three regions, hospital types and activity levels broadens representation but does not eliminate concerns about national generalizability.

Moreover, as physician practices were self-reported in Questionnaire B and were not independently verified, social-desirability bias may have been introduced, and thus potentially overestimating the rate of VL use.

Additionally, because responses to Questionnaire B were anonymous, respondents’ centers could not be identified. Consequently, within-center clustering could not be accounted for, and responses were treated as independent observations. This should be acknowledged as a limitation of this study.

Furthermore, given the cross-sectional design of this study, no casual relationships can be established. The analysis aimed only to identify statistical associations between VL use and demographic, institutional, and educational factors.

Finally, the relatively small absolute number of respondents reduced the statistical power of the study. Consequently, the number of statistical tests was deliberately limited to minimize the risk of inflating the type I error rate.

## Conclusions

Reported VL availability was high among responding Belgian MICUs. However, in the absence of Belgian national recommendations specific to prehospital laryngoscope choice, reported VL use in clinical practice remains uncommon among responding MICU physicians. Reported indications for VL use vary widely, and protocols regarding VL use indications are inconsistent across participating centers. These findings provide the first insights into VL availability and use in the Belgian prehospital setting.
